# Antibacterial and Antibiotic-Modifying Activity of Methanol Extracts from Six Cameroonian Food Plants against Multidrug-Resistant Enteric Bacteria

**DOI:** 10.1155/2017/1583510

**Published:** 2017-08-20

**Authors:** Joachim K. Dzotam, Victor Kuete

**Affiliations:** Department of Biochemistry, Faculty of Science, University of Dschang, Dschang, Cameroon

## Abstract

The present work was designed to investigate the antibacterial activities of methanol extracts from six Cameroonian edible plants and their synergistic effects with some commonly used antibiotics against multidrug-resistant (MDR) Gram-negative bacteria expressing active efflux pumps. The extracts were subjected to qualitative phytochemical screening and the microdilution broth method was used for antibacterial assays. The results of phytochemical tests indicate that all tested crude extracts contained polyphenols, flavonoids, triterpenes, and steroids. Extracts displayed selective antibacterial activities with the minimal inhibitory concentration (MIC) values ranging from 32 to 1024 *μ*g/mL. The lowest MIC value (32 *μ*g/mL) was recorded with* Coula edulis* extract against* E. coli* AG102 and* K. pneumoniae* K2 and with* Mangifera indica* bark extract against* P. aeruginosa* PA01 and* Citrus sinensis* extract against* E. coli* W3110 which also displayed the best MBC (256 *μ*g/mL) value against* E. coli* ATCC8739. In combination with antibiotics, extracts from* M. indica* leaves showed synergistic effects with 75% (6/8) of the tested antibiotics against more than 80% of the tested bacteria. The findings of the present work indicate that the tested plants may be used alone or in combination in the treatment of bacterial infections including the multidrug-resistant bacteria.

## 1. Introduction

The discovery of antibiotics has inevitably fostered the development and emergence of resistances in bacteria, regardless of the mechanism of action of the antibiotic involved. For decades, and in order to reduce the development and epidemic spread of resistance, physicians and scientists have called for appropriate use of antibiotics [1]. Prudent use of antibiotics in humans demands that physicians establish that a bacterial infection is responsible for the patient's symptoms before an antibiotic prescription is made. By contrast, in agriculture, antibiotics are used in the absence of acute infection [2]. Bacteria faced with this fierce struggle against them have developed several mechanisms of resistance against antimicrobial agents whose main ones include enzymatic inactivation [3], modification of the drug target(s), and reduction of intracellular drug concentration by changes in membrane permeability or by the overexpression of efflux pumps [4]. With respect to efflux pumps, they provide a self-defense mechanism by which antibiotics are actively removed from the cell. For antibacterials, this results in sublethal drug concentrations at the active site that in turn may predispose the organism to the development of high-level target-based resistance [5]. In this way, efflux pumps inevitably become targets for the research and/or development of new, less toxic, and effective molecules capable, alone or in combination with the usual antibiotics, of effectively fighting infections involving multidrug-resistant pathogens. Medicinal plants in general and food plants in particular have been used for centuries to cure ailments of man. Today, there is a real revival of interest in these almost-exploited sources [6] of molecules whose pharmacological efficacy is no longer to be demonstrated [7]. The present work was designed to investigate the in vitro ability of methanol extracts from six Cameroonian edible plants (*Psidium guajava* Linn.,* Persea americana* Mill.,* Camellia sinensis* Linn.,* Mangifera indica* Linn.,* Coula edulis* Baill., and* Citrus sinensis* Linn.), to potentiate the activity of some commonly used antibiotics vis-à-vis Gram-negative multidrug-resistant bacteria.

## 2. Material and Methods

### 2.1. Plant Material and Extraction

The plant materials used in this work were collected in the period of March to April 2015 in two regions of Cameroon and included leaves and bark of* M. indica*; fruits, leaves, and bark* of P. guajava*; leaves and bark of* P. americana* collected at Koung-Khi division (West Region); leaves of* C. sinensis* and the fruits of* C. sinensis* collected at Menoua division (West Region); and nuts of* C. edulis* collected at Mungo division (Littoral Region). The plants were identified at the National Herbarium (Yaounde, Cameroon) where voucher specimens were deposited under the reference numbers (Table 1). Each plant sample was cleaned and air-dried and the powder (300 g) was extracted with methanol (MeOH, 1 L) for 48 h at room temperature. The extract was then concentrated under reduced pressure to give residues which constituted the crude extract. All extracts were then kept at 4°C until further use.

### 2.2. Preliminary Phytochemical Investigations

The major phytochemical classes such as alkaloids (Dragendorff's and Mayer's tests), triterpenes (Liebermann-Burchard's test), flavonoids (aluminum chloride test), anthraquinones (Borntrager's test), polyphenols (ferric chloride test), sterols (Salkowski's test), coumarins (lactone test), saponins (foam test), and tannins (gelatin test) (Table 2) were investigated according to the commonly described phytochemical methods [43, 44].

### 2.3. Bacteria Strains and Culture Media

The studied bacteria strains included reference (from American Type Culture Collection) and clinical (Laboratory collection) strains of* Escherichia coli*,* Enterobacter aerogenes*,* Klebsiella pneumoniae*,* Providencia stuartii*, and* Pseudomonas aeruginosa*. Their bacterial features were previously reported [45, 46]. They were maintained on agar slant at 4°C and subcultured on a fresh appropriate agar plates 24 h prior to any antimicrobial test. Mueller-Hinton Agar (MHA) was used for the activation of bacteria, while Mueller-Hinton Broth (MHB) was used for the MIC determinations.

### 2.4. Chemicals for Antimicrobial Assays

The reference antibiotics used in the present work included tetracycline (TET), cefepime (CEP), ciprofloxacin (CIP), streptomycin (STP), chloramphenicol (CHL), ampicillin (AMP), erythromycin (ERY), and kanamycin (KAN), all provided by Sigma-Aldrich, St Quentin Fallavier, France.* p*-Iodonitrotetrazolium (INT) chloride and phenylalanine-arginine-ß-naphthylamide (PAßN) were used as microbial growth indicator and efflux pumps inhibitor (EPI), respectively [47].

### 2.5. Bacterial Susceptibility Determination

Rapid INT colorimetric assay [47] with some modifications [48] was used to determine the minimal inhibitory concentrations (MIC) of the plant extracts and those of reference antibiotics (RA) against the studied bacteria. The test samples and RA were first of all dissolved in DMSO/Mueller-Hinton Broth (MHB). The final concentration of DMSO was lower than 2.5% and did not affect the microbial growth [49]. The solution obtained was then added to Mueller-Hinton Broth and serially diluted twofold (in a 96-well microplate). One hundred microliters (100 *μ*L) of inoculum 1.5 × 10^6^ CFU/mL prepared in appropriate broth was then added [48, 50]. The plates were covered with a sterile plate sealer, then agitated to mix the contents of the wells using a plate shaker, and incubated at 37°C for 18 h. The assay was repeated thrice. Wells containing adequate broth, 100 *μ*L of inoculum, and DMSO to a final concentration of 2.5% served as negative control. The MIC of samples was detected after 18 h incubation at 37°C, following addition (40 *μ*L) of 0.2 mg/mL of INT and incubation at 37°C for 30 min. Viable bacteria reduced the yellow dye to pink. MIC was defined as the sample concentration that prevented the color change of the medium and exhibited complete inhibition of microbial growth [47]. The MBC was determined by adding 50 *μ*L aliquots of the preparations, which did not show any growth after incubation during MIC assays, to 150 *μ*L of adequate broth. These preparations were incubated at 37°C for 48 h. The MBC was regarded as the lowest concentration of extract, which did not produce a color change after addition of INT as mentioned above [50].

The role of efflux pumps in the susceptibility of Gram-negative bacteria to* M. indica*,* C. edulis*,* C. sinensis*, leaves from* P. guajava*,* P. americana*, and* C. sinensis*, and crude extracts was evaluated by testing them in the presence of an efflux pump inhibitor (EPI), the PA*β*N (at 30 *μ*g/mL) against fifteen selected MDR phenotypes (*E. coli* AG100A, AG102, and AG100ATet,* E. aerogenes* EA289, EA27, EA298, and CM64,* K. pneumoniae* KP55 and KP63,* E. cloacae* BM47, BM67, and ECCI69,* P. aeruginosa* PA124, and* P. stuartii* NAE16 and PS2636).

To evaluate the potentiating effect of tested extracts, a preliminary combination at their subinhibitory concentrations (MIC/2, MIC/4, MIC/8, and MIC/16) with antibiotics was assessed against* P. aeruginosa* PA124 strain (see Table S1 in Supplementary Material available online at https://doi.org/10.1155/2017/1583510). The appropriate subinhibitory concentrations were then selected on the basis of their ability to improve the activity of the maximum antibiotic [51, 52]. These subinhibitory concentrations for selected extracts were further tested in combination with antibiotics against more MDR bacteria. The fractional inhibitory concentration (FIC) of each combination was then calculated as the ratio of MIC of antibiotic in combination versus MIC of antibiotic alone (MIC_Antibiotic  in  combination_/MIC_Antibiotic  alone_) and the interpretation made was as follows: synergistic (≤0.5), indifferent (1 to 4), or antagonistic (>4) [53, 54]. All assays were performed in triplicate.

## 3. Results

### 3.1. Phytochemical Composition of Plant's Extracts

The results of the phytochemical screening (Table 2) showed that all the tested plant's extracts contain polyphenols, flavonoids, triterpenes, and sterols. The other classes of secondary metabolites were selectively distributed. Also, the extract from the leaves and bark of* P. americana* as well as those of* M. indica* and the extract from* C. edulis* nuts contain all the classes of screened secondary metabolites.

### 3.2. Antibacterial Activity of the Extracts

The antibacterial activities of plants' extracts alone and in some cases, in the presence of the PA*β*N on a panel of 29 Gram-negative bacteria, was investigated (Table 3). It appears that* P. guajava* leaves extract inhibited the growth of all (100%) the tested bacteria, and the recorded MICs ranged from 256 to 1024 *μ*g/ml. Other samples showed very selective activity; their inhibitory activities were recorded on 26/29 (89.65%;* P. guajava* bark* and P. americana* leaves and bark), 25/29 (82.75%;* C. sinensis*), 23/29 (79.31%;* C. edulis*), 22/29 (75.86%;* C. sinensis*), 21/29 (72.41%;* M. indica* leaves and bark), and 11/29 (37.93%;* P. guajava* fruits). The lowest MIC value of 32 *μ*g/mL was recorded with the crude extract of* C. edulis* against* E. coli* AG102 and* K. pneumoniae* K2, as well as with the extract from bark of* M. indica* against* P. aeruginosa* PA01 and the extract of* C. sinensis* against* E. coli* W3110. Extract from* C. sinensis* displayed the best MBC (256 *μ*g/mL) value against* E. coli* ATCC8739.

### 3.3. Role of Efflux Pumps in the Susceptibility of Gram-Negative Bacteria to the Tested Plant Extracts

Fifteen of the studied MDR bacteria were also tested for their susceptibility to plant extracts in the presence of the PA*β*N at 30 *μ*g/ml. The results showed that when combined with extracts, PA*β*N improves the activity (decrease of MIC values) of* C. sinensis* on 11/15 (73.33%) tested MDR strains. EPI also improved the activity of other extracts against the selected bacteria (Table 3).

### 3.4. Effects of the Association of Extracts with Antibiotics

A preliminary study performed against* P. aeruginosa* PA124 allowed us to select six out of ten extracts at the appropriate subinhibitory concentrations of MIC/2 and MIC/4 for further studies. All the six extracts were combined separately to eight antibiotics (CIP, STP, CHL, ERY, KAN, TET, CEF, and AMP) to evaluate their possible synergetic effects. The results summarized in Tables 4–9 showed that the synergic effects were noted with all the tested extracts with most of the tested antibiotics except *β*-lactams (CEF and AMP). At MIC/2, the extract from* M. indica* leaves showed synergistic effects with 7 of the 8 antibiotics (CIP, STP, CHL, ERY, KAN, TET, and AMP) (87.5%) against at least 70% MDR bacteria for each antibiotic. At that same MIC,* C. sinensis* extract improved the activity of all (8/8) tested antibiotics against at least 50% of the tested MDR bacteria. Synergies with FIC ranging from 0.5 to <0.007 were noted with the associations of each of the seven extracts with antibiotics. These low FIC values obtained in the majority of cases (FIC < 0.007 for associations of* M. indica* + *β*-lactams vis-à-vis almost all the bacterial strains and also with many other extracts with streptomycin against many bacterial strains) indicate that extracts have potentiated the activity of antibiotics vis-à-vis the studied bacteria.

## 4. Discussion

*Chemical Composition of the Tested Plant Extracts*. Phytochemical screening revealed the presence of several classes of secondary metabolites such as alkaloids, polyphenols, flavonoids, anthraquinones, coumarins, saponins, tannins, triterpenes, and steroids. Several in vitro experiments proved that molecules belonging to these classes were found active on pathogenic microorganisms [55].

*Antibacterial Activity of the Tested Extracts*. After several decades of antibiotic use, pathogenic bacteria of human and animal origin have reached alarming levels of resistance. The reduced efficacy and increasing toxicity of synthetic drugs further aggravate this problem. Scientists are thus directed to seek more active natural or organic molecules [14]. It is imperative in developing countries that effective and less expensive antibacterials should be developed to accommodate all patients, regardless of financial status, in order to eliminate some human factors that can cause MDR. Medicinal plants have known to possess antimicrobial properties against MDR phenotypes [48–50, 56–59] and differences observed between antibacterial activities of the extracts in the present study could be due to the differences in the composition as well as the mechanism of action of their bioactive compounds [55, 60]. Different solvents extracts of leaves from* P. guajava* have been separately documented to be inhibitory against both Gram-positive and Gram-negative bacteria [14, 15, 61]. In the present study, methanol extract from* P. guajava* leaf demonstrated significant inhibitory activities against both sensitive and MDR bacteria, confirming the previous reports on its antimicrobial activity. According to Kuete and Efferth [62], the antibacterial activity of a plant extract is considered significant when MIC values are below 100 *μ*g/mL, moderate when 100 ≤ MICs ≤ 625 *μ*g/mL, and weak when MICs > 625 *μ*g/mL. Consequently, the methanol* P. guajava* leaf extract tested herein had moderate activity. Besides, the activity (MIC of 32 and 64 *μ*g/mL) observed with* C. edulis* nuts and* M. indica* bark as well as* C. sinensis* leaves extracts against* E. coli* AG102,* P. aeruginosa* PA01, and* K. pneumoniae* K2, respectively, can be considered important. The antibacterial activities of mango (*M. indica*) extracts against some microorganisms (Gram-positive and Gram-negative bacteria and* Candida albicans* yeast) have already been demonstrated [38, 63] and it is thought that the antibacterial activity of mango extract is due to the presence of gallotannin and mangiferin [64]. Different extracts of* C. sinensis* having antimicrobial activity were reported against Gram-positive and Gram-negative bacteria and fungi [65, 66] and it is thought that their antibacterial activities are due to the presence of catechin. In fact, catechin particularly affects the membrane fluidity in both hydrophilic and hydrophobic regions of lipid bilayers of the microorganism [67] or inhibits the action of DNA polymerases [68]. The present study provides additional data on the ability of this plant to fight MDR bacteria.

The leaves of* P. americana* are widely used for ethnomedicinal purposes worldwide. In order to confirm its multiple medicinal properties, a study conducted by Nathaniel et al. [18] showed that the methanolic extract from leaves of* P. americana* had good antimicrobial activity against enteric microorganisms (Gram-negative and Gram-positive bacteria and also yeasts). This is in accordance with the present study as we also found that methanol extracts of both leaves and bark of the plant exhibited similar activities against several MDR bacteria, with leaves' extract having good inhibitor effects (64 *μ*g/mL) against the* E. coli* AG102 strain. Considering the fact that some of the tested plants are used as food with limited toxicity, the overall activity recorded with several extracts, most notably those of* C. sinensis*,* C. sinensis*, and* C. edulis* or the fruits from* P. guajava*, could be considered important. However, a threshold for significant antibacterial effects has been proposed for MIC values ranging within 100–512 *μ*g/mL when samples tested are edible parts of plants [69].

*Role of Efflux Pumps in the Susceptibility of Gram-Negative Bacteria to the Tested Extracts*. The studied bacterial strains used in this work mostly resist by efflux mechanism, which consists in expelling all toxic compounds (including antibiotics) out of the cytoplasm, thus preventing them from reaching their intracellular targets [70]. These efflux pumps can however be blocked competitively or not, in order to restore not only the intracellular concentration but also the activity of antibacterials acting on intracellular targets [71]. In the presence of PA*β*N (EPI), the antibacterial activity of* C. sinensis* extract was improved on 11/15 (73.37%) tested MDR strains. However, the antibacterial activities of other extracts were also improved on few selected bacteria. These results suggest that these extracts (*C. sinensis* especially) have an intracellular targets.

*Effects of Association of Extracts with Antibiotics*. The reduced efficiency of known antibiotics propels many researchers to search for novel compounds, as well as molecules capable of improving or at least restoring the efficiency of available antibiotics against resistant bacteria. The combination of both compounds can then help to prevent the emergence of resistant mutants and enable restoring the activity of antibiotics vis-à-vis resistant strains. Synergies with FIC ranging from 0.5 to <0.007 were noted with the associations of each of the extracts and antibiotics on at least one tested MDR bacteria. The extracts from leaves of* M. indica* and* C. sinensis* and also that from peels of* C. sinensis* had important synergistic effects with most of the tested antibiotics against the majority of bacteria. These results suggest that extracts inhibited the efflux pumps, increasing the activity of these antibiotics [72, 73]. Few cases of indifference (1 ≤ FIC < 4) were found in the majority of associations; meanwhile antagonistic effect was obtained when* C. edulis* extract was combined with streptomycin against* E. aerogenes* EA-CM64 strain. All plants tested in the present study (Table 1) are used traditionally to treat several affections or diseases with symptoms related to microbial infections [8–10, 16, 17, 19, 20, 27, 29, 40], and data obtained therefore consolidate their traditional use. The presence of previously reported secondary metabolites in the tested plants [11, 12, 18, 21, 22, 27, 30, 31, 40, 41] (Table 1) is also in accordance with the present studies and could in part justify their antibacterial effects. Natural compounds belonging to terpenoids (for example, camosic acid and isopimarane derivatives), flavonoids (for example, 5′-methoxyhydnocarpin) as well as alkaloids (for example, pheophorbide A or reserpine) and others have been identified as efflux pumps inhibitors [74, 75]. Extract from* M. indica* and* C. sinensis* having the best synergistic effects contain all these classes of secondary metabolites, probably explaining their antibiotic potentiation effects. These results could be explained by the interaction between active compounds of extracts and the active ingredients of antibiotics.

## 5. Limitations

Our study has limitations. It mainly reports the activity of crude plant extracts and the identification of the active constituents of the plant would be necessary for better understanding of the reported effects. Though this study deals with food plants, the cytotoxicity studies in normal cell lines will be performed to ascertain their safety.

## 6. Conclusion

The results of this study can provide some explanations on the traditional use of certain parts of the plants tested herein to combat infections, particularly those caused by enteric bacteria. Evidence of the antibacterial activity of the studied plants, especially edible ones, as well as the ability of some of them to improve the activity of commonly used antibiotics, is an indication that there is a possibility of discovering alternative antibacterials in these plants.

## Supplementary Material

Table S 1. Preliminary test of MIC of different antibiotics after the association of the extracts at MIC/2, MIC/4, MIC/8 and MIC/16 against *P. aeruginosa* PA124 strain.

## Figures and Tables

**Table 1:** Plants used in the present study and evidence of their bioactivities.

Species (family); voucher number^*∗*^	Traditional uses	Parts used traditionally	Bioactive or potentially bioactive components	Bioactivity of crude extract
*Psidium guajava *Linn. (Myrtaceae); 2884/SRF/Cam	To treat wounds, lesions, ulcers, diarrhea, cholera, hypertension, and obesity and the control of diabetes mellitus [8]. Anti-inflammatory effect, cough, diabetes, kidney problems, diarrhea, tonic, laxative, antihelminthic [9], conjunctivitis, oral care, and antispasmodic [10].	Leaves, fruits, flowers, bark, and roots	Tannins, flavonoids (myricetin, quercetin, luteolin and kaempferol), essential oils (caryophyllene, nerolidol, *β*-bisabolene, aromadendrene, p-selinene, *α*-pinene and 1,8-cineol), triterpenoids (oleanic acid, ursolic acid, catecholic acid, guayavolic acid, maslinic acid, ellagic acid) and *β*-sitosterol [11]; essential oil: propylbenzene, allyl benzene, and so forth [12].	*Antibacterial activity of essential oil*: *Sa, Sf, Lspp, Esa, Aspp, Ec, Pv, Ea, St, Pa, andKp* [12]; *antioxidant activity of leaves essential oil* [13]. *Ethanolic extract of leaves*: *Ec, Kp, Pa, Sa, Ab,* MRSA, and VRE [14]; antiproliferative, antiseptic and antifungal activity [9]; *ethanolic extract of leaves*: antioxidant, antitumor, and antibacterial (against *Sm*, *Smt*, and *Sor*) [15].

*Persea americana* Mill. (Lauraceae); 57756/HNC	Anticancer activity, the food industry has grown tremendous interest in processing this crop and enhancing its value [16]. *P. americana *leaves' extracts have been used as analgesic, anti-inflammatory, hypoglycaemic, anticonvulsant, antidiabetic, and vasorelaxant [17].	Leaves, fruit, flowers, bark, and roots	*Crude leaves extracts*: glycosides, alkaloids, tannins, saponins, flavonoids, terpenoids, and steroids [18].	*Methanol, chloroform, ethyl acetate, and petroleum ether leaves extracts* were tested against *Ec, St, Sa, Ca, Pa*,and* Bs; *antioxidant activity [18]; *aqueous leaves' extract*: anti-inflammatory and analgesic activity [17].

*Citrus sinensis* Linn. (Rutaceae); 25859/HNC	Treat ailments like constipation, cramps, colic, diarrhea, bronchitis, tuberculosis, cough, cold, obesity, menstrual disorder, angina, hypertension, anxiety, depression, and stress [19]; used to soothe sore throats, indigestion, relieve intestinal gas and bloating and resolve phlegm and as an additive for flavoring to our foods [20].	Leaves, flowers, seeds, peel, fruits, and barks	Polyphenols (caffeic acid, p-coumaric acid, ferulic acid, and sinapinic acid), flavonoids (hesperidin, narirutin, naringin, eriocitrin) [21], essential oil composition: D-limonene, *β*-myrcene, *α*-pinene, *β*-pinene, *γ*-terpinene, *α*-terpinolene, *α*-caryophyllene, copaene, *β*-phellandrene [22] and many other constituents.	*Ethanolic extracts of peels and juice* (Q):* Sta, Sau, Sm, Ss,Kp*, and *Ec* [20]; *ethanolic extracts of peels*: *Sta*, *Pv, Kp, Ec, Bs*, and *Pa* [23]; *crude extract of juice* (Q): *Ec*, *Sta*, and MRSA [24]; *crude extracts from different parts of the plants* reduce high blood pressure, respiratory problems, rheumatism [25, 26], and anticancer, antimicrobial, and antioxidant activity [22].

*Coula edulis *Baill. (Olacaceae); African walnut 22931 SRF/Cam	The stomachic bark decoction is used to treat dysentery in Liberia and the bark powder decoction is used in equatorial Africa to stimulate appetite and counteract anemia [27].	Leaves, stem bark, fruits, and roots	*Ethanolic extract of leaves, stem bark and roots*: alkaloids, flavonoids, tannins, saponins, terpenes, and cardiac glycosides [27].	*Ethanolic extracts of bark and leaves*: *Pa*,* Sa, Ec, St, *and *Ca *[27]. *Methanolic extract of the bark*: antiplasmodial activity [28].

*Mangifera indica *Linn. (Anacardiaceae); 18646/HNC	Anti-syphilitic, vulnerary, antiemetic, anti-inflammatory, cough, hiccup, hyperdipsia, burning sensation, hemorrhages, haemoptysis, hemorrhoids, wounds, ulcers, diarrhoea, dysentery, pharyngopathy, scorpion string, wounds, ulcers, anorexia, and dyspepsia [29].	Leaves, flowers, barks, roots, stones, and fruits	Carotenoids (provitamin A compound, beta-carotene, lutein, and alpha-carotene), polyphenols [30] such as quercetin, kaempferol, gallic acid, caffeic acid, catechins, tannins, and mangiferin [31].	*Mangiferin*: anticancer (breast, renal, colon and leukemia cancer cell lines [32, 33]; antidiabetic [34]; antimalarial [35]; *methanol and aqueous extracts*: *Sa, Sp, Pa, Ca*, and *Ef* [36]; *Se, Lm, Ec* [37]; *ethanol and benzene extracts*: *Pv, Pf, Sfx, Kp*, and *St* [38]; antiamoebic [39].

*Camellia sinensis *Linn. (Theaceae); 43103/HNC	Aiding digestion, blood purification, ensuring regularity, lowering body temperature, strengthening teeth and bones, boosting immune system, enhancing heart function, suppressing aging, deterring food poisoning, fighting virus, and lowering blood sugar levels [40].	Leaves, stem, bark, and roots	Polyphenols (catechins and their derivatives), methylxanthines (caffeine, theophylline and theobromine), carbohydrates, proteins, free amino acids, vitamins (vitamin C and carotenoids), volatile compounds, lipids, chlorophylls, saponins and inorganic elements (fluorine, manganese and aluminum) [40, 41].	*Methanolic extract of leaves*: antioxidant activity. These antioxidant properties are beneficial for several chronic diseases related to oxidative stress, including cancer and cardiovascular and neurodegenerative diseases [42].

^*∗*^HNC: Herbier National du Cameroun; SRF/Cam: Société des Réserves Forestières du Cameroun; *Sm*:* Streptococcus mutans*;* Smt*:* Streptococcus mitis*;* Sor*:* Streptococcus oralis*;* Sta*:* Staphylococcus aureus*;* Sau*:* Staphylococcus auricularis*;* Ss*:* Streptococcus salivarius*;* Sp*:* Streptococcus pneumoniae*;* Kp*:* Klebsiella pneumoniae; Sa*: *Streptococcus aureus*;* Ec*:* Escherichia coli*;* Se*:* Salmonella enteritidis*;* Bs*:* Bacillus subtilis*;* St*:* Salmonella typhi*;* Ef*:* Enterococcus faecalis*;* Sf: Staphylococcus faecalis*;* Pv: Proteus vulgaris*;* MRSA: *methicillin-resistant *Staphylococcus aureus*;* Pf*:* Pseudomonas fluorescens*;* Sfx*:* Shigella flexneri*;* Pa*:* Pseudomonas aeruginosa*;* Ca*:* Candida albicans*;* Ea*:* Enterobacter aerogenes*;* Asp*:* Acinetobacter *spp.;* VRE*:vancomycin-resistant* Enterococcus*;* Lspp*:* Lactobacillus spp.*;* Ab*:* Acinetobacter baumannii*;* Esa*:* Enterococcus aerogenes*;* Lm*:* Listeria monocytogenes*.

**Table 2:** Extraction yields, aspects, and phytochemical composition of the plant extracts.

Extracts	*Psidium guajava*			*Persea americana*		*Citrus sinensis*	*Coula edulis*	*Mangifera indica*		*Camellia sinensis*
Parts used	Leaves	Bark	Fruits	Leaves	Bark	Peel	Fruits	Leaves	Bark	Leaves
Yield^*∗*^ (%)	5.47	4.17	6.12	5.15	4.47	6.56	7.25	5.36	4.26	5.8
Alkaloids	+	+	−	+	+	+	+	+	+	+
Polyphenols	+	+	+	+	+	+	+	+	+	+
Flavonoids	+	+	+	+	+	+	+	+	+	+
Anthraquinones	−	−	−	+	+	−	+	+	+	−
Coumarins	+	+	+	+	+	+	+	+	+	−
Tannins	+	+	−	+	+	+	+	+	+	+
Triterpenes	+	+	+	+	+	+	+	+	+	+
Sterols	+	+	+	+	+	+	+	+	+	+
Saponins	+	+	−	+	+	+	+	+	+	+

−: absent; +: present; ^*∗*^yield calculated as the ratio of the mass of the obtained methanol extract/mass of the plant powder.

**(a):** 

Bacterial strains	Tested samples and MIC and MBC and MIC in the presence of PA*β*N in parenthesis (*μ*g/mL)														
	*P. guajava (leaves)*			*P. guajava (fruits)*			*P. guajava (bark)*			*P. americana (leaves)*			*P. americana (bark)*		
	*MIC*	*MBC*	*R*	*MIC*	*MBC*	*R*	*MIC*	*MBC*	*R*	*MIC*	*MBC*	*R*	*MIC*	*MBC*	*R*
*Escherichia coli*															
*ATCC8739*	512	—	—	1024	—	—	1024	—	—	256	—	—	256	—	—
*ATCC10536*	512	—	—	1024	—	—	512	—	—	128	—	—	256	—	—
*AG100*	1024	—	—	1024	—	—	512	—	—	256	—	—	256	—	—
*AG100A*	256 (256)	—	—	512	—	—	1024	—	—	256 (256)	—	—	512 (512)	—	—
*AG102*	512 (512)	—	—	256	1024	4	1024	—	—	**64** (16)	512	8	128 (64)	1024	8
*AG100ATet*	512 (128)	—	—	—	—	—	1024	—	—	512 (128)	—	—	512 (128)	—	—
*MC4100*	512	—	—	—	—	—	1024	—	—	128	—	—	512	—	—
*W3110*	1024	—	—	—	—	—	1024	—	—	256	—	—	256	—	—

*Enterobacter aerogenes*															
*ATCC13048*	256	—	—	1024	—	—	512	—	—	512	—	—	512	—	—
*EA-CM64*	512 (512)	—	—	—	—	—	1024	—	—	512 (512)	—	—	512 (512)	—	—
*EA3*	512	—	—	—	—	—	512	—	—	256	—	—	128	—	—
*EA27*	128 (128)	—	—	—	—	—	—	—	—	128 (256)	—	—	256 (256)	—	—
*EA289*	256 (128)	—	—	—	—	—	512	—	—	256 (256)	—	—	512 (512)	—	—
*EA294*	1024	—	—	—	—	—	—	—	—	256	—	—	128	1024	8
*EA298*	1024 (1024)	—	—	—	—	—	—	—	—	128 (128)	1024	*8*	256 (256)	1024	4

*Klebsiella pneumoniae*															
*ATCC11296*	1024	—	—	—	—	—	—	—	—	512	—	—	1024	—	—
*K2*	512	—	—	1024	—	—	1024	—	—	256	—	—	256	—	—
*K24*	512	—	—	—	—	—	1024	—	—	512	—	—	512	—	—
*KP55*	1024 (1024)	—	—	1024	—	—	512	—	—	— (—)	—	—	— (—)	—	—
*KP63*	256 (16)	—	—	—	—	—	512	—	—	512 (<8)	—	—	128 (<8)	1024	8

*Providencia stuartii*															
*ATCC29916*	512	—	—	—	—	—	1024	—	—	—	—	—	—	—	—
*ATCC299645*	512	—	—	1024	—	—	1024	—	—	512	—	—	1024	—	—
*PS2636*	512 (512)	—	—	512	1024	*2*	512	—	—	256 (256)	1024	*4*	512 (512)	—	—
*NEA16*	1024 (1024)	—	—	—	—	—	512	1024	*2*	1024 (512)	—	—	256 (16)	1024	*4*

*Enterobacter cloacae*															
*BM47*	256 (1024)	—	—	—	—	—	512	—	—	512 (512)	—	—	128 (128)	—	—
*BM67*	256 (256)	—	—	1024	—	—	512	—	—	512 (512)	—	—	256 (256)	—	—
*ECCI69*	512 (512)	—	—	—	—	—	512	—	—	1024 (1024)	—	—	— (—)	—	—

*Pseudomonas aeruginosa*															
*PA01*	512	—	—	—	—	—	1024	—	—	—	—	—	128	—	—
*PA124*	1024 (1024)	—	—	—	—	—	1024	—	—	— (—)	—	—	1024 (512)	—	—

*R*: MIC/MBC; —: MIC > 1024 or not detected; ( ): values in parenthesis are MIC of substance in the presence of PA*β*N at 30 *μ*g/mL; values in bold are the best MIC values for the plant extracts.

**(b):** 

Bacterial strains	Tested samples and MIC and MBC and MIC in the presence of PA*β*N in parenthesis (*μ*g/mL)														
	*Citrus sinensis*			*M. indica (leaves)*			*M. indica (bark)*			*Camellia sinensis*			*Coula edulis*		
	*MIC*	*MBC*	*R*	*MIC*	*MBC*	*R*	*MIC*	*MBC*	*R*	*MIC*	*MBC*	*R*	*MIC*	*MBC*	*R*
*Escherichia coli*															
*ATCC8739*	**64**	256	4	—	*—*	*—*	256	*—*	*—*	512	*—*	*—*	**64**	256	4
*ATCC10536*	256	—	—	512	*—*	*—*	256	*—*	*—*	512	*—*	*—*	**64**	512	8
*AG100*	128	—	—	1024	*—*	*—*	—	*—*	*—*	512	*—*	*—*	**64**	*—*	*—*
*AG100A*	512 (256)	*—*	—	1024	*—*	*—*	— (—)	*—*	*—*	512 (256)	*—*	*—*	1024	*—*	*—*
*AG102*	128 (<8)	1024	4	1024 (1024)	*—*	*—*	1024 (1024)	*—*	*—*	256 (256)	*—*	*—*	**32**	256	8
*AG100ATet*	512 (8)	*—*	—	1024 (1024)	*—*	*—*	— (—)	*—*	*—*	256 (256)	*—*	*—*	1024	*—*	*—*
*MC4100*	**64**	*—*	—	1024	*—*	*—*	1024	*—*	*—*	1024	*—*	*—*	**64**	*—*	*—*
*W3110*	**32**	512	16	1024	*—*	*—*	1024	*—*	*—*	1024	*—*	*—*	**64**	*—*	*—*

*Enterobacter aerogenes*															
*ATCC13048*	512	1024	2	512	*—*	*—*	512	*—*	*—*	512	*—*	*—*	512	*—*	*—*
*EA-CM64*	— (16)	*—*	—	— (1024)	*—*	*—*	— (—)	*—*	*—*	— (—)	*—*	*—*	**64**	*—*	*—*
*EA3*	256	*—*	—	1024	*—*	*—*	512	*—*	*—*	1024	*—*	*—*	128	*—*	*—*
*EA27*	512 (256)	*—*	—	512 (256)	*—*	*—*	512 (256)	*—*	*—*	1024 (1024)	*—*	*—*	256	*—*	*—*
*EA289*	512 (<8)	*—*	—	— (—)	*—*	*—*	512 (—)	*—*	*—*	512 (64)	*—*	*—*	512	*—*	*—*
*EA294*	128	512	4	—	*—*	*—*	—	*—*	*—*	1024	*—*	*—*	**64**	1024	16
*EA298*	128 (128)	512	4	— (—)	*—*	*—*	— (—)	*—*	*—*	512 (512)	*—*	*—*	256	*—*	*—*

*Klebsiella pneumoniae*															
*ATCC11296*	512	*—*	*—*	—	*—*	*—*	—	*—*	*—*	512	*—*	*—*	*—*	*—*	*—*
*K2*	128	*—*	*—*	1024	*—*	*—*	1024	*—*	*—*	**64**	*—*	*—*	**32**	*—*	*—*
*K24*	*—*	*—*	*—*	1024	*—*	*—*	1024	*—*	*—*	512	*—*	*—*	1024	*—*	*—*
*KP55*	— (—)	*—*	*—*	1024 (1024)	*—*	*—*	1024 (1024)	*—*	*—*	— (—)	*—*	*—*	1024	*—*	*—*
*KP63*	256 (<8)	*—*	*—*	1024 (1024)	*—*	*—*	512 (256)	*—*	*—*	256 (64)	*—*	*—*	512	*—*	*—*

*Providencia stuartii*															
*ATCC29916*	*—*	*—*	*—*	512	*—*	*—*	1024	*—*	*—*	512	*—*	*—*	*—*	*—*	*—*
*ATCC299645*	*—*	*—*	*—*	1024	*—*	*—*	—	*—*	*—*	512	*—*	*—*	128	1024	8
*PS2636*	1024 (<8)	*—*	*—*	512 (512)	*—*	*—*	512 (512)	*—*	*—*	— (—)	*—*	*—*	512	512	1
*NEA16*	128 (16)	1024	8	512 (512)	*—*	*—*	512 (512)	*—*	*—*	1024 (1024)	1024	*1*	128	1024	8

*Enterobacter cloacae*															
*BM47*	256 (16)	*—*	*—*	512	*—*	*—*	512 (512)	*—*	*—*	1024 (1024)	*—*	*—*	1024	*—*	*—*
*BM67*	512 (512)	*—*	*—*	1024	*—*	*—*	512 (512)	*—*	*—*	256 (256)	1024	4	*—*	*—*	*—*
*ECCI69*	512 (512)	*—*	*—*	— (—)	*—*	*—*				512 (256)	—	*—*	*—*	*—*	*—*

*Pseudomonas aeruginosa*															
*PA01*	128	512	4	1024	*—*	*—*	**32**	*—*	*—*	512	*—*	*—*	*—*	*—*	*—*
*PA124*	— (1024)	*—*	*—*	1024 (512)	*—*	*—*	512 (512)	*—*	*—*	1024 (1024)	*—*	*—*	*—*	*—*	*—*

*R*: CMB/CMI; —: MIC > 1024 or not detected; ( ): values in parenthesis are MIC of substance in the presence of PA*β*N at 30 *μ*g/mL; values in bold are the best MIC values for the plant extracts.

**(c):** 

Bacterial strains	*Chloramphenicol*		
	MIC	MBC	*R*
*Escherichia coli*			
*ATTC8739*	4	16	4
*ATCC10536*	4	128	32
*AG100*	8	64	8
*AG100A*	64	—	—
*AG102*	64	256	4
*AG100ATet*	128	—	—
*MC4100*	8	64	8
*W3110*	8	256	32

*Enterobacter aerogenes*			
*ATCC13048*	8	64	8
*EA-CM64*	128	—	—
*EA3*	32	256	8
*EA27*	128	—	—
*EA289*	256	—	—
*EA294*	8	128	16
*EA298*	32	64	2

*Klebsiella pneumoniae*			
*ATCC11296*	64	—	—
*K2*	32	128	4
*K24*	128	256	2
*KP55*	64	128	2
*KP63*	64	256	4

*Providencia stuartii*			
*ATCC29916*	128	—	—
*ATCC299645*	64	256	4
*PS2636*	128	—	—
*NEA16*	32	256	8

*Enterobacter cloacae*			
*BM47*	256	—	—
*BM67*	128	—	—
*ECCI69*	—	256	—

*Pseudomonas aeruginosa*			
*PA01*	32	—	—
*PA124*	256	—	—

*R*: CMB/CMI; —: MIC > 1024 or not detected; values in parenthesis are MIC of substance in the presence of PA*β*N at 30 *μ*g/mL; values in bold are the best MIC values for the plant extracts.

**Table 4:** MIC of different antibiotics after the association of the extract of *Citrus sinensis* at MIC/2, MIC/4 against ten MDR bacteria strains.

Antibiotics^a^	Bacterial strains and MIC (*μ*g/mL) of antibiotics in the absence and presence of the extract^b^											
	Extract concentration	PA124	AG102	AG100Atet	EA289	CM64	KP55	KP63	NEA16	PS2636	BM47	PBSS^c^ (%)
CHL	0	128	8	128	256	256	32	32	16	64	128	
	MIC/2	**32** (**0**.**25)**^S^	<**2** (<**0**.**25**)^S^	**32** (**0**.**25**)^S^	256 (1)^I^	**64 **(**0**.**25**)^S^	32 (1)^I^	**4 **(**0**.**125**)^S^	16 (1)^I^	**16 **(**0**.**25**)^S^	128 (1)^I^	**60**
	MIC/4	128 (1)^I^	<**2 **(<**0**.**25**)^S^	128 (1)^I^	256 (1)^I^	**128 **(**0**.**5**)^S^	32 (1)^I^	**4 **(**0**.**125**)^S^	16 (1)^I^	64 (1)^I^	128 (1)^I^	30

CIP	0	64	2	>64	>64	>64	2	<0.5	4	8	<0.5	
	MIC/2	**16 **(**0**.**25**)^S^	**1 **(**0**.**5**)^S^	>64 (≥1)^I^	**64 **(<**1**)^S^	**16 **(<**0**.**25**)^S^	**1 **(**0**.**5**)^S^	<0.5 (**≤**1)^I^	4 (1)^I^	<**1 **(<**0**.**125**)^S^	<0.5 (**≤**1)^I^	**60**
	MIC/4	**32 **(**0**.**5**)^S^	**1 **(**0**.**5**)^S^	>64 (≥1)^I^	>64 (≥1)^I^	**16 **(<**0**.**25**)^S^	**1 **(**0**.**5**)^S^	<0.5 (**≤**1)^I^	4 (1)^I^	**2 **(**0**.**25**)^S^	<0.5 (**≤**1)^I^	**50**

TET	0	16	16	32	64	4	32	2	4	2	32	
	MIC/2	**4 **(**0**.**25**)^S^	<**1 **(<**0**.**062**)^S^	64 (2)^I^	64 (1)^I^	**1 **(**0**.**25**)^S^	32 (1)^I^	2 (1)^I^	4 (1)^I^	<**1 **(<**0**.**5**)^S^	32 (1)^I^	40
	MIC/4	**8 **(**0**.**5**)^S^	<**1 **(<**0**.**062**)^S^	64 (2)^I^	64 (1)^I^	**1 **(**0**.**25**)^S^	32 (1)^I^	2 (1)^I^	4 (1)^I^	<**1 **(<**0**.**5**)^S^	32 (1)^I^	40

ERY	0	128	8	128	256	8	16	4	16	16	16	
	MIC/2	128 (1)^I^	<**2 **(<**0**.**25**)^S^	128 (1)^I^	256 (1)^I^	8 (1)^I^	16 (1)^I^	<**2 **(<**0**.**5**)^S^	16 (1)^I^	**8 **(**0**.**5**)^S^	16 (1)^I^	30
	MIC/4	128 (1)^I^	<**2 **(<**0**.**25**)^S^	128 (1)^I^	256 (1)^I^	8 (1)^I^	16 (1)^I^	<**2 **(<**0**.**5**)^S^	16 (1)^I^	16 (1)^I^	16 (1)^I^	20

KAN	0	128	4	8	32	32	32	<2	16	16	8	
	MIC/2	128 (1)^I^	<**2 **(<**0**.**5**)^S^	8 (1)^I^	**16 **(**0**.**5**)^S^	**16 **(**0**.**5**)^S^	32 (1)^I^	<2 (**≤**1)^I^	**8 **(**0**.**5**)^S^	16 (1)^I^	8 (1)^I^	**50**
	MIC/4	128 (1)^I^	<**2 **(<**0**.**5**)^S^	8 (1)^I^	**16 **(**0**.**5**)^S^	**16 **(**0**.**5**)^S^	32 (1)^I^	<2 (**≤**1)^I^	**8 **(**0**.**5**)^S^	16 (1)^I^	8 (1)^I^	**50**

STP	0	256	>256	>256	128	8	64	8	16	>256	64	
	MIC/2	**32 **(**0**.**125**)^S^	**32 **(≥**0**.**125**)^S^	<**2 **(**≤0**.**007**)^S^	128 (1)^I^	<**2 **(**0**.**25**)^S^	**16 **(**0**.**25**)^S^	<**2 **(<**0**.**25**)^S^	16 (1)^I^	<**2 **(**≤0**.**007**)^S^	**32 **(**0**.**5**)^S^	**80**
	MIC/4	**128 **(**0**.**5**)^S^	**128 **(≥**0**.**5**)^S^	<**2 **(**≤0**.**007**)^S^	128 (1)^I^	8 (1)^I^	**16 **(**0**.**25**)^S^	<**2 **(<**0**.**25**)^S^	16 (1)^I^	**4 **(≥**0**.**062**)^S^	64 (1)^I^	**60**

AMP	0	>256	>256	>256	>256	>256	>256	>256	>256	>256	>256	
	MIC/2	>256 (≥1)^I^	<**2 **(≤**0**.**007**)^S^	>256 (≥1)^I^	>256 (≥1)^I^	<**2 **(**≤0**.**007**)^S^	>256 (≥1)^I^	<**2 **(**≤0**.**007**)^S^	<**2 **(**≤0**.**007**)^S^	<**2 **(**≤0**.**007**)^S^	>256 (≥1)^I^	**50**
	MIC/4	>256 (≥1)^I^	<**2 **(**≤0**.**007**)^S^	>256 (≥1)^I^	>256 (≥1)^I^	<**2 **(**≤0**.**007**)^S^	>256 (≥1)^I^	**256 **(<**1**)^S^	<**2 **(**≤0**.**007**)^S^	<**2 **(**≤0**.**007**)^S^	>256 (≥1)^I^	**50**

CEF	0	>256	>256	>256	>256	>256	>256	>256	>256	>256	>256	
	MIC/2	>256 (≥1)^I^	>256 (≥1)^I^	>256 (≥1)^I^	>256 (≥1)^I^	>256 (≥1)^I^	>256 (≥1)^I^	<**2 **(**≤0**.**007**)^S^	>256 (≥1)^I^	>256 (≥1)^I^	>256 (≥1)^I^	10
	MIC/4	>256 (≥1)^I^	>256 (≥1)^I^	>256 (≥1)^I^	>256 (≥1)^I^	>256 (≥1)^I^	>256 (≥1)^I^	**256 **(<**1**)^S^	>256 (≥1)^I^	>256 (≥1)^I^	>256 (≥1)^I^	10

^a^Antibotics [TET: tetracycline, CIP: ciprofloxacin, STP: streptomycin, KAN: kanamycin, CHL: chloramphenicol, ERY: erythromycin, AMP: ampicillin, and CEF: cefepime]. ^b^Bacterial strains: *Escherichia coli* [AG102, AG100Atet], *Pseudomonas aeruginosa* [PA124], *Enterobacter aerogenes* [CM64, EA27, EA289], *Klebsiella pneumonia* [KP55, KP63], *Providencia stuartii* [NAE16], and *Enterobacter cloacae* [BM47]. ^c^PBSS: percentage of bacteria strain on which synergism has been observed. Values in parenthesis are fractional inhibitory concentration (FIC) of the antibiotics after association with plants extract; S: synergy; I: indifference. The values in bold represent the cases of synergy between extract and antibiotic.

**Table 5:** MIC of different antibiotics after the association of the extract of *Coula edulis* at MIC/2, MIC/4 against ten MDR bacteria strains.

Antibiotics^a^	Bacterial strains and MIC (*μ*g/mL) of antibiotics in the absence and presence of the extract^b^											
	Extract concentration	PA124	AG102	AG100Atet	EA289	CM64	KP55	KP63	NEA16	PS2636	BM47	PBSS^c^ (%)
CHL	0	128	8	128	256	256	32	32	16	64	128	
	MIC/2	**64** (**0**.**5**)^S^	<**2** (<**0**.**25**)^S^	128 (1)^I^	256 (1)^I^	**64** (**0**.**25**)^S^	32 (1)^I^	32 (1)^I^	16 (1)^I^	**8** (**0**.**125**)^S^	128 (1)^I^	40
	MIC/4	**64** (**0**.**5**)^S^	**4** (**0**.**5**)^S^	128 (1)^I^	256 (1)^I^	**128** (**0**.**5**)^S^	32 (1)^I^	32 (1)^I^	16 (1)^I^	**8** (**0**.**125**)^S^	128 (1)^I^	40

CIP	0	64	2	>64	>64	>64	2	<0.5	4	8	<0.5	
	MIC/2	**16** (**0**.**25**)^S^	<**0**.**5** (<**0**.**25**)^S^	>64 (≥1)^I^	**32** (<**0**.**5**)^S^	**64** (<**1**)^S^	2 (1)^I^	<0.5 (≤1)^I^	<**0**.**5** (<**0**.**25**)^S^	<**0**.**5** (<**0**.**125**)^S^	<0.5 (≤1)^I^	**60**
	MIC/4	**16** (**0**.**25**)^S^	<**0**.**5** (<**0**.**25**)^S^	>64 (≥1)^I^	**32** (<**0**.**5**)^S^	**64** (<**1**)^S^	2 (1)^I^	<0.5 (≤1)^I^	<**0**.**5** (<**0**.**25**)^S^	<**0**.**5** (<**0**.**125**)^S^	<0.5 (≤1)^I^	**60**

TET	0	16	16	32	64	4	32	2	4	2	32	
	MIC/2	16 (1)^I^	<**1** (<**0**.**062**)^S^	64 (2)^I^	64 (1)^I^	<**0**.**5** (<**0**.**25**)^S^	32 (1)^I^	2 (1)^I^	4 (1)^I^	<**1** (<**0**.**5**)^S^	32 (1)^I^	30
	MIC/4	16 (1)^I^	<**1** (<**0**.**062**)^S^	64 (2)^I^	64 (1)^I^	<**0**.**5** (<**0**.**25**)^S^	32 (1)^I^	2 (1)^I^	4 (1)^I^	<**1** (<**0**.**5**)^S^	32 (1)^I^	20

ERY	0	128	8	128	256	8	16	4	16	16	16	
	MIC/2	128 (1)^I^	<**2** (<**0**.**25**)^S^	128 (1)^I^	256 (1)^I^	16 (2)^I^	16 (1)^I^	4 (1)^I^	**8** (**0**.**5**)^S^	**8** (**0**.**5**)^S^	16 (1)^I^	30
	MIC/4	128 (1)^I^	<**2** (<**0**.**25**)^S^	128 (1)^I^	256 (1)^I^	16 (2)^I^	16 (1)^I^	4 (1)^I^	**8** (**0**.**5**)^S^	16 (1)^I^	16 (1)^I^	20

KAN	0	128	4	8	32	32	32	<2	16	16	8	
	MIC/2	128 (1)^I^	<**2** (<**0**.**5**)^S^	8 (1)^I^	32 (1)^I^	32 (1)^I^	32 (1)^I^	<2 (≤1)^I^	16 (1)^I^	16 (1)^I^	8 (1)^I^	0
	MIC/4	128 (1)^I^	<**2** (<**0**.**5**)^S^	8 (1)^I^	32 (1)^I^	32 (1)^I^	32 (1)^I^	<2 (≤1)^I^	16 (1)^I^	16 (1)^I^	8 (1)^I^	20

STP	0	256	>256	>256	128	8	64	8	16	>256	64	
	MIC/2	**64** (**0**.**25**)^S^	**8** (<**0**.**031**)^S^	<**2** (≤**0**.**007**)^S^	128 (1)^I^	128 (16)^A^	64 (1)^I^	8 (1)^I^	16 (1)^I^	**8** (<**0**.**031**)^S^	64 (1)^I^	40
	MIC/4	**64** (**0**.**25**)^S^	>256 (≥1)^I^	**64** (<**0**.**25**)^S^	128 (1)^I^	128 (16)^A^	64 (1)^I^	8 (1)^I^	16 (1)^I^	**128** (<**0**.**5**)^S^	64 (1)^I^	30

AMP	0	>256	>256	>256	>256	>256	>256	>256	>256	>256	>256	
	MIC/2	**256** (<**1**)^S^	<**2** (≤**0**.**007**)^S^	>256 (≥1)^I^	>256 (≥1)^I^	>256 (≥1)^I^	>256 (≥1)^I^	>256 (≥1)^I^	<**2** (≤**0**.**007**)^S^	<**2** (≤**0**.**007**)^S^	>256 (≥1)^I^	40
	MIC/4	>256 (≥1)^I^	<**2** (≤**0**.**007**)^S^	>256 (≥1)^I^	>256 (≥1)^I^	>256 (≥1)^I^	>256 (≥1)^I^	>256 (≥1)^I^	<**2** (≤**0**.**007**)^S^	<**2** (≤**0**.**007**)^S^	>256 (≥1)^I^	30

CEF	0	>256	>256	>256	>256	>256	>256	>256	>256	>256	>256	
	MIC/2	>256 (≥1)^I^	>256 (≥1)^I^	>256 (≥1)^I^	>256 (≥1)^I^	>256 (≥1)^I^	>256 (≥1)^I^	>256 (≥1)^I^	>256 (≥1)^I^	>256 (≥1)^I^	>256 (≥1)^I^	00
	MIC/4	>256 (≥1)^I^	>256 (≥1)^I^	>256 (≥1)^I^	>256 (≥1)^I^	>256 (≥1)^I^	>256 (≥1)^I^	>256 (≥1)^I^	>256 (≥1)^I^	>256 (≥1)^I^	>256 (≥1)^I^	00

^a^Antibotics [TET: tetracycline, CIP: ciprofloxacin, STP: streptomycin, KAN: kanamycin, CHL: chloramphenicol, ERY: erythromycin, AMP: ampicillin, and CEF: cefepime]. ^b^Bacterial strains: *Escherichia coli* [AG102, AG100Atet], *Pseudomonas aeruginosa* [PA124], *Enterobacter aerogenes* [CM64, EA27, EA289], *Klebsiella pneumonia* [KP55, KP63], *Providencia stuartii* [NAE16], and *Enterobacter cloacae* [BM47]. ^c^PBSS: percentage of bacteria strain on which synergism has been observed. Values in parenthesis are fractional inhibitory concentration (FIC) of the antibiotics after association with plants extract; S: synergy; I: indifference; A: antagonism. The values in bold represent the cases of synergy between extract and antibiotic.

**Table 6:** MIC of different antibiotics after the association of the extract of leaves from *Mangifera indica* at MIC/2, MIC/4 against ten MDR bacteria strains.

Antibiotics^a^	Bacterial strains and MIC (*μ*g/mL) of antibiotics in the absence and presence of the extract^b^											
	Extract concentration	PA124	AG102	AG100Atet	EA289	CM64	KP55	KP63	NEA16	PS2636	BM47	PBSS^c^ (%)
CHL	0	128	8	128	256	256	32	32	16	64	128	
	MIC/2	**32** (**0**.**25**)^S^	<**2** (<**0**.**25**)^S^	**4** (**0**.**031**)^S^	**64** (**0**.**25**)^S^	**128** (**0**.**5**)^S^	**4** (**0**.**125**)^S^	**8** (**0**.**25**)^S^	**4** (**0**.**25**)^S^	**16** (**0**.**25**)^S^	**32** (**0**.**25**)^S^	**100**
	MIC/4	**32** (**0**.**25**)^S^	**4** (**0**.**5**)^S^	**4** (**0**.**031**)^S^	**128** (**0**.**5**)^S^	**128** (**0**.**5**)^S^	32 (1)^I^	32 (1)^I^	**8** (**0**.**5**)^S^	64 (1)^I^	128 (1)^I^	**60**

CIP	0	64	2	>64	>64	>64	2	<0.5	4	8	<0.5	
	MIC/2	**32** (**0**.**5**)^S^	<**0**.**5** (<**0**.**25**)^S^	**8** (<**0**.**125**)^S^	**2** (<**0**.**031**)^S^	**64** (<**1**)^S^	2 (1)^I^	<0.5 (≥1)^I^	<**0**.**5** (<**0**.**125**)^S^	<**0**.**5** (<**0**.**062**)^S^	<0.5 (≥1)^I^	**70**
	MIC/4	**32** (**0**.**5**)^S^	<**0**.**5** (<**0**.**25**)^S^	>64 (≥1)^I^	**2** (<**0**.**031**)^S^	**64** (<**1**)^S^	2 (1)^I^	<0.5 (≥1)^I^	**2** (**0**.**5**)^S^	**4** (**0**.**5**)^S^	<0.5 (≥1)^I^	**60**

TET	0	16	16	32	64	4	32	2	4	2	32	
	MIC/2	32 (2)^I^	**4** (**0**.**25**)^S^	**8** (**0**.**25**)^S^	<**2** (<**0**.**031**)^S^	**2** (**0**.**5**)^S^	**8** (**0**.**25**)^S^	**1** (**0**.**5**)^S^	<**0**.**5** (<**0**.**125**)^S^	<**0**.**5** (<**0**.**25**)^S^	**8** (**0**.**25**)^S^	**90**
	MIC/4	32 (2)^I^	**8** (**0**.**5**)^S^	**16** (**0**.**5**)^S^	**16** (**0**.**25**)^S^	**2** (**0**.**5**)^S^	**8** (**0**.**25**)^S^	**1** (**0**.**5**)^S^	<**0**.**5** (<**0**.**125**)^S^	<**0**.**5** (<**0**.**25**)^S^	**16** (**0**.**5**)^S^	**90**

ERY	0	128	8	128	256	8	16	4	16	16	16	
	MIC/2	128 (1)^I^	<**2** (<**0**.**25**)^S^	**32** (**0**.**25**)^S^	**32** (**0**.**125**)^S^	**4** (**0**.**5**)^S^	**8** (**0**.**5**)^S^	<**2** (<**0**.**5**)^S^	**4** (**0**.**25**)^S^	<**2** (<**0**.**125**)^S^	<**2** (<**0**.**125**)^S^	**90**
	MIC/4	128 (1)^I^	<**2** (<**0**.**25**)^S^	**32** (**0**.**25**)^S^	**128** (**0**.**5**)^S^	**4** (**0**.**5**)^S^	16 (1)^I^	<**2** (<**0**.**5**)^S^	**8** (**0**.**5**)^S^	**4** (**0**.**25**)^S^	**4** (**0**.**25**)^S^	**80**

KAN	0	128	4	8	32	32	32	<2	16	16	8	
	MIC/2	128 (1)^I^	<**2** (<**0**.**5**)^S^	**4** (**0**.**5**)^S^	<**2** (<**0**.**062**)^S^	**8** (**0**.**25**)^S^	**4** (**0**.**125**)^S^	<2 (≥1)^I^	<**2** (<**0**.**125**)^S^	**4** (**0**.**25**)^S^	8 (1)^I^	**70**
	MIC/4	128 (1)^I^	<**2** (<**0**.**5**)^S^	8 (1)^I^	**8** (**0**.**25**)^S^	**16** (**0**.**5**)^S^	**4** (**0**.**125**)^S^	<2 (≥1)^I^	**8** (**0**.**5**)^S^	16 (1)^I^	8 (1)^I^	**50**

STP	0	256	>256	>256	128	8	64	8	16	>256	64	
	MIC/2	**128** (**0**.**5**)^S^	**16** (<**0**.**062**)^S^	**16** (<**0**.**062**)^S^	**32** (**0**.**25**)^S^	**4** (**0**.**5**)^S^	**32** (**0**.**5**)^S^	**4** (**0**.**5**)^S^	**4** (**0**.**25**)^S^	**64** (<**0**.**5**)^S^	**4** (**0**.**062**)^S^	**100**
	MIC/4	**128** (**0**.**5**)^S^	**32** (<**0**.**125**)^S^	**16** (<**0**.**062**)^S^	**64** (**0**.**5**)^S^	8 (1)^I^	**32** (**0**.**5**)^S^	8 (1)^I^	16 (1)^I^	>256 (na)	**8** (**0**.**125**)^S^	**60**

AMP	0	>256	>256	>256	>256	>256	>256	>256	>256	>256	>256	
	MIC/2	**256** (<**1**)^S^	**128** (<**0**.**5**)^S^	**128** (<**0**.**5**)^S^	**128** (<**0**.**5**)^S^	**128** (<**0**.**5**)^S^	**256** (<**1**)^S^	**8** (<**0**.**031**)^S^	**256** (<**1**)^S^	**128** (<**0**.**5**)^S^	>256 (≥1)^I^	**90**
	MIC/4	>256 (≥1)^I^	**256** (<**1**)^S^	**256** (<**1**)^S^	**256** (<**1**)^S^	**256** (<**1**)^S^	>256 (≥1)^I^	>256 (≥1)^I^	>256 (≥1)^I^	>256 (≥1)^I^	>256 (≥1)^I^	40

CEF	0	>256	>256	>256	>256	>256	>256	>256	>256	>256	>256	
	MIC/2	**256** (<**1**)^S^	**256** (<**1**)^S^	>256 (≥1)^I^	**256** (<**1**)^S^	**128** (<**0**.**5**)^S^	>256 (≥1)^I^	>256 (≥1)^I^	**256** (<**1**)^S^	>256 (≥1)^I^	>256 (≥1)^I^	**50**
	MIC/4	>256 (≥1)^I^	>256 (≥1)^I^	>256 (≥1)^I^	>256 (≥1)^I^	**256** (<**1**)^S^	>256 (≥1)^I^	>256 (≥1)^I^	>256 (≥1)^I^	>256 (≥1)^I^	>256 (≥1)^I^	10

^a^Antibotics [TET: tetracycline, CIP: ciprofloxacin, STP: streptomycin, KAN: kanamycin, CHL: chloramphenicol, ERY: erythromycin, AMP: ampicillin, and CEF: cefepime]. ^b^Bacterial strains: *Escherichia coli* [AG102, AG100Atet], *Pseudomonas aeruginosa* [PA124], *Enterobacter aerogenes* [CM64, EA27, EA289], *Klebsiella pneumonia* [KP55, KP63], *Providencia stuartii* [NAE16], and *Enterobacter cloacae* [BM47]. ^c^PBSS: percentage of bacteria strain on which synergism has been observed. Values in parenthesis are fractional inhibitory concentration (FIC) of the antibiotics after association with plants extract; S: synergy; I: indifference. The values in bold represent the cases of synergy between extract and antibiotic.

**Table 7:** MIC of different antibiotics after the association of the extract of leaves from *Psidium guajava* at MIC/2, MIC/4 against ten MDR bacteria strains.

Antibiotics^a^	Bacterial strains and MIC (*μ*g/mL) of antibiotics in the absence and presence of the extract^b^											
	Concentration of the extract	PA124	AG102	AG100Atet	EA289	CM64	KP55	KP63	NEA16	PS2636	BM47	PBSS^c^ (%)
CHL	0	128	8	128	256	256	32	32	16	64	128	
	MIC/2	**32** (**0**.**25**)^S^	**4** (**0**.**5**)^S^	**64** (**0**.**5**)^S^	256 (1)^I^	256 (1)^I^	32 (1)^I^	**16** (**0**.**5**)^S^	**8** (**0**.**5**)^S^	**32** (**0**.**25**)^S^	**32** (**0**.**25**)^S^	**70**
	MIC/4	**32** (**0**.**25**)^S^	8 (1)^I^	128 (1)^I^	256 (1)^I^	256 (1)^I^	32 (1)^I^	32 (1)^I^	16 (1)^I^	64 (1)^I^	128 (1)^I^	10

CIP	0	64	4	>64	>64	>64	2	<0.5	4	8	<0.5	
	MIC/2	**32** (**0**.**5**)^S^	4 (1)^I^	>64 (≥1)^I^	**16** (<**0**.**25**)^S^	**64** (<**1**)^S^	2 (1)^I^	<0.5 (≥1)^I^	**2** (**0**.**5**)^S^	**1** (**0**.**125**)^S^	<0.5 (≥1)^I^	**50**
	MIC/4	**32** (**0**.**5**)^S^	4 (1)^I^	>64 (≥1)^I^	>64 (≥1)^I^	**64** (<**1**)^S^	2 (1)^I^	<0.5 (≥1)^I^	4 (1)^I^	**4** (**0**.**5**)^S^	<0.5 (≥1)^I^	30

TET	0	16	16	32	64	4	32	2	4	2	32	
	MIC/2	32 (2)^I^	**4** (**0**.**25**)^S^	32 (1)^I^	**32** (**0**.**5**)^S^	4 (1)^I^	**16** (**0**.**5**)^S^	<**0**.**5** (<**0**.**25**)^S^	<**0**.**5** (<**0**.**125**)^S^	<**0**.**5** (<**0**.**25**)^S^	**16** (**0**.**5**)^S^	**70**
	MIC/4	32 (2)^I^	16 (1)^I^	32 (1)^I^	**32** (**0**.**5**)^S^	4 (1)^I^	32 (1)^I^	**1** (**0**.**5**)^S^	4 (1)^I^	<**0**.**5** (<**0**.**25**)^S^	32 (1)^I^	30

ERY	0	128	8	128	256	8	16	4	16	16	16	
	MIC/2	128 (1)^I^	**4** (**0**.**5**)^S^	128 (1)^I^	**128** (**0**.**5**)^S^	<**2** (<**0**.**25**)^S^	16 (1)^I^	**2** (**0**.**5**)^S^	16 (1)^I^	16 (1)^I^	**16** (**0**.**5**)^S^	**50**
	MIC/4	128 (1)^I^	**4** (**0**.**5**)^S^	128 (1)^I^	**128** (**0**.**5**)^S^	<**2** (<**0**.**25**)^S^	16 (1)^I^	4 (1)^I^	16 (1)^I^	16 (1)^I^	16 (1)^I^	10

KAN	0	128	4	8	32	32	32	<2	16	16	8	
	MIC/2	128 (1)^I^	<**2** (<**0**.**5**)^S^	8 (1)^I^	**4** (**0**.**5**)^S^	32 (1)^I^	**8** (**0**.**25**)^S^	<2 (≥1)^I^	**8** (**0**.**5**)^S^	**4** (**0**.**25**)^S^	8 (1)^I^	**50**
	MIC/4	128 (1)^I^	<**2** (<**0**.**5**)^S^	8 (1)^I^	32 (1)^I^	32 (1)^I^	32 (1)^I^	<2 (≥1)^I^	**8** (**0**.**5**)^S^	16 (1)^I^	8 (1)^I^	20

STP	0	256	>256	>256	128	8	64	8	16	>256	64	
	MIC/2	**128** (**0**.**5**)^S^	**16** (<**0**.**062**)^S^	**128** (<**0**.**5**)^S^	128 (1)^I^	**4** (**0**.**5**)^S^	64 (1)^I^	**4** (**0**.**5**)^S^	16 (1)^I^	**128** (<**0**.**5**)^S^	**16** (**0**.**25**)^S^	**70**
	MIC/4	**128** (**0**.**5**)^S^	**128** (<**0**.**5**)^S^	**256** (<**1**)^S^	128 (1)^I^	8 (1)^I^	64 (1)^I^	8 (1)^I^	16 (1)^I^	>256 (≥1)^I^	**16** (**0**.**25**)^S^	40

AMP	0	>256	>256	>256	>256	>256	>256	>256	>256	>256	>256	
	MIC/2	**256** (<**1**)^S^	>256 (≥1)^I^	>256 (≥1)^I^	**256** (<**1**)^S^	**128** (<**0**.**5**)^S^	>256 (≥1)^I^	**128** (<**0**.**5**)^S^	>256 (≥1)^I^	**256** (<**1**)^S^	>256 (≥1)^I^	50
	MIC/4	>256 (≥1)^I^	>256 (≥1)^I^	>256 (≥1)^I^	>256 (≥1)^I^	>256 (≥1)^I^	>256 (≥1)^I^	>256 (≥1)^I^	>256 (≥1)^I^	>256 (≥1)^I^	>256 (≥1)^I^	10

CEF	0	>256	>256	>256	>256	>256	>256	>256	>256	>256	>256	
	MIC/2	**256** (<**1**)^S^	>256 (≥1)^I^	>256 (≥1)^I^	>256 (≥1)^I^	>256 (≥1)^I^	>256 (≥1)^I^	>256 (≥1)^I^	>256 (≥1)^I^	>256 (≥1)^I^	>256 (≥1)^I^	10
	MIC/4	>256 (≥1)^I^	>256 (≥1)^I^	>256 (≥1)^I^	>256 (≥1)^I^	>256 (≥1)^I^	>256 (≥1)^I^	>256 (≥1)^I^	>256 (≥1)^I^	>256 (≥1)^I^	>256 (≥1)^I^	00

^a^Antibotics [TET: tetracycline, CIP: ciprofloxacin, STP: streptomycin, KAN: kanamycin, CHL: chloramphenicol, ERY: erythromycin, AMP: ampicillin, and CEF: cefepime]. ^b^Bacterial strains: *Escherichia coli* [AG102, AG100Atet], *Pseudomonas aeruginosa* [PA124], *Enterobacter aerogenes* [CM64, EA27, EA289], *Klebsiella pneumonia* [KP55, KP63], *Providencia stuartii* [NAE16], and *Enterobacter cloacae* [BM47]. ^c^PBSS: percentage of bacteria strain on which synergism has been observed. Values in parenthesis are fractional inhibitory concentration (FIC) of the antibiotics after association with plants extract; S: synergy; I: indifference. The values in bold represent the cases of synergy between extract and antibiotic.

**Table 8:** MIC of different antibiotics after the association of the extract of *Camellia sinensis* at MIC/2, MIC/4 against ten MDR bacteria strains.

Antibiotics^a^	Bacterial strains and MIC (*μ*g/mL) of antibiotics in the absence and presence of the extract^b^											
	Extract concentration	PA124	AG102	AG100Atet	EA289	CM64	KP55	KP63	NEA16	PS2636	BM47	PBSS^c^ (%)
CHL	0	128	8	128	256	256	32	32	16	64	128	
	MIC/2	**8** (**0**.**0625**)^S^	<**2** (<**0**.**25**)^S^	**64** (**0**.**5**)^S^	**128** (**0**.**5**)^S^	**64** (**0**.**25**)^S^	**8** (**0**.**25**)^S^	**16** (**0**.**5**)^S^	<**2** (<**0**.**125**)^S^	**4** (**0**.**25**)^S^	**16** (**0**.**25**)^S^	**100**
	MIC/4	**8** (**0**.**0625**)^S^	<**2** (<**0**.**25**)^S^	**64** (**0**.**5**)^S^	256 (1)^I^	**128** (**0**.**5**)^S^	**16** (**0**.**5**)^S^	**16** (**0**.**5**)^S^	**8** (**0**.**5**)^S^	64 (1)^I^	128 (1)^I^	**70**

CIP	0	64	4	>64	>64	>64	2	<0.5	4	8	<0.5	
	MIC/2	**32** (**0**.**5**)^S^	<**0**.**5** (<**0**.**125**)^S^	**16** (<**0**.**25**)^S^	>64 (≥1)^I^	**16** (<**0**.**25**)^S^	<**0**.**5** (<**0**.**25**)^S^	<0.5 (≥1)^I^	<**0**.**5** (<**0**.**125**)^S^	<**0**.**5** (<**0**.**062**)^S^	<0.5 (≥1)^I^	**70**
	MIC/4	**32** (**0**.**5**)^S^	<**0**.**5** (<**0**.**125**)^S^	>64 (≥1)^I^	>64 (≥1)^I^	**16** (<**0**.**25**)^S^	<**0**.**5** (<**0**.**25**)^S^	<0.5 (≥1)^I^	4 (1)^I^	**2** (**0**.**25**)^S^	<0.5 (≥1)^I^	**50**

TET	0	16	16	32	64	4	32	2	4	2	32	
	MIC/2	16 (1)^I^	<**1** (<**0**.**062**)^S^	**8** (**0**.**25**)^S^	**16** (**0**.**25**)^S^	**2** (**0**.**5**)^S^	32 (1)^I^	**1** (**0**.**5**)^S^	<**0**.**5** (<**0**.**125**)^S^	<**0**.**5** (<**0**.**25**)^S^	**16** (**0**.**5**)^S^	**80**
	MIC/4	16 (1)^I^	<**1** (<**0**.**062**)^S^	**16** (**0**.**5**)^S^	**32** (**0**.**5**)^S^	4 (1)^I^	32 (1)^I^	2 (1)^I^	<**0**.**5** (<**0**.**125**)^S^	<**0**.**5** (<**0**.**25**)^S^	32 (1)^I^	**50**

ERY	0	128	8	128	256	8	16	4	16	16	16	
	MIC/2	128 (1)^I^	<**2** (<**0**.**25**)^S^	**32** (**0**.**25**)^S^	**64** (**0**.**25**)^S^	<**2** (<**0**.**125**)^S^	16 (1)^I^	4 (1)^I^	**4** (**0**.**25**)^S^	**4** (**0**.**25**)^S^	<**2** (<**0**.**125**)^S^	**70**
	MIC/4	128 (1)^I^	**4** (**0**.**5**)^S^	**64** (**0**.**5**)^S^	**64** (**0**.**25**)^S^	<**2** (<**0**.**125**)^S^	16 (1)^I^	4 (1)^I^	**8** (**0**.**5**)^S^	**4** (**0**.**25**)^S^	**4** (**0**.**25**)^S^	**70**

KAN	0	128	4	8	32	32	32	<2	16	16	8	
	MIC/2	**64** (**0**.**5**)^S^	<**2** (<**0**.**5**)^S^	**4** (**0**.**5**)^S^	**16** (**0**.**5**)^S^	**8** (**0**.**25**)^S^	32 (1)^I^	<2 (≥1)^I^	<**0**.**5** (<**0**.**031**)^S^	16 (1)^I^	8 (1)^I^	**60**
	MIC/4	**64** (**0**.**5**)^S^	<**2** (<**0**.**5**)^S^	8 (1)^I^	**16** (**0**.**5**)^S^	**16** (**0**.**5**)^S^	32 (1)^I^	<2 (≥1)^I^	**8** (**0**.**5**)^S^	16 (1)^I^	8 (1)^I^	**50**

STP	0	256	>256	>256	128	8	64	8	16	>256	64	
	MIC/2	**64** (**0**.**25**)^S^	**32** (<**0**.**125**)^S^	**128** (<**0**.**5**)^S^	**64** (**0**.**5**)^S^	**4** (**0**.**5**)^S^	64 (1)^I^	<**2** (<**0**.**25**)^S^	<**2** (<**0**.**125**)^S^	**64** (<**0**.**25**)^S^	**4** (**0**.**0625**)^S^	**90**
	MIC/4	**64** (**0**.**25**)^S^	**64** (<**0**.**25**)^S^	**128** (<**0**.**5**)^S^	**64** (**0**.**5**)^S^	8 (1)^I^	64 (1)^I^	**8** (**1**)^I^	**8** (**0**.**5**)^S^	**64** (<**0**.**25**)^S^	**16** (**0**.**25**)^S^	**70**

AMP	0	>256	>256	>256	>256	>256	>256	>256	>256	>256	>256	
	MIC/2	>256 (≥1)^I^	**128** (<**0**.**5**)^S^	**128** (<**0**.**5**)^S^	**64** (<**0**.**25**)^S^	**128** (<**0**.**5**)^S^	>256 (≥1)^I^	**64** (<**0**.**25**)^S^	**128** (<**0**.**5**)^S^	>256 (≥1)^I^	**128** (<**0**.**5**)^S^	**70**
	MIC/4	>256 (≥1)^I^	**128** (<**0**.**5**)^S^	>256 (≥1)^I^	**256** (<**1**)^S^	>256 (≥1)^I^	>256 (≥1)^I^	>256 (≥1)^I^	>256 (≥1)^I^	>256 (≥1)^I^	>256 (≥1)^I^	20

CEF	0	>256	>256	>256	>256	>256	>256	>256	>256	>256	>256	
	MIC/2	>256 (≥1)^I^	>256 (≥1)^I^	>256 (≥1)^I^	**128** (<**0**.**5**)^S^	>256 (≥1)^I^	>256 (≥1)^I^	**128** (<**0**.**5**)^S^	**256** (<**0**.**5**)^S^	**128** (<**0**.**5**)^S^	**128** (<**0**.**5**)^S^	**50**
	MIC/4	>256 (≥1)^I^	>256 (≥1)^I^	>256 (≥1)^I^	>256 (≥1)^I^	>256 (≥1)^I^	>256 (≥1)^I^	>256 (≥1)^I^	>256 (≥1)^I^	>256 (≥1)^I^	>256 (≥1)^I^	00

^a^Antibotics [TET: tetracycline, CIP: ciprofloxacin, STP: streptomycin, KAN: kanamycin, CHL: chloramphenicol, ERY: erythromycin, AMP: ampicillin, and CEF: cefepime]. ^b^Bacterial strains: *Escherichia coli* [AG102, AG100Atet], *Pseudomonas aeruginosa* [PA124], *Enterobacter aerogenes* [CM64, EA27, EA289], *Klebsiella pneumonia* [KP55, KP63], *Providencia stuartii* [NAE16], and *Enterobacter cloacae* [BM47]. ^c^PBSS: percentage of bacteria strain on which synergism has been observed. Values in parenthesis are fractional inhibitory concentration (FIC) of the antibiotics after association with plants extract; S: synergy; I: indifference; A: antagonism. The values in bold represent the cases of synergy between extract and antibiotic.

**Table 9:** M1IC of different antibiotics after the association of the extract of bark from *Persea americana *at MIC/2, MIC/4 against ten MDR bacteria strains.

Antibiotics^a^	Bacterial strains and MIC (*μ*g/mL) of antibiotics in the absence and presence of the extract^b^											
	Extract concentration	PA124	AG102	AG100Atet	EA289	CM64	KP55	KP63	NEA16	PS2636	BM47	PBSS^c^ (%)
CHL	0	128	8	128	256	256	32	32	16	64	128	
	MIC/2	**64** (**0.5**)^S^	**4** (**0.5**)^S^	128 (1)^I^	256 (1)^I^	256 (1)^I^	32 (1)^I^	**16** (**0.5**)^S^	16 (1)^I^	64 (1)^I^	**64** (**0.5**)^S^	40
	MIC/4	**64** (**0.5**)^S^	8 (1)^I^	128 (1)^I^	256 (1)^I^	256 (1)^I^	32 (1)^I^	**16** (**0.5**)^S^	16 (1)^I^	64 (1)^I^	128 (1)^I^	20

CIP	0	64	8	>64	>64	>64	2	<0.5	4	8	<0.5	
	MIC/2	**32** (**0.5**)^S^	**4** (**0.5**)^S^	>64 (≥1)^I^	>64 (≥1)^I^	**32** (<**0.5**)^S^	<**0.5** (<**0.25**)^S^	<0.5 (≥1)^I^	4 (1)^I^	8 (1)^I^	<0.5 (≥1)^I^	40
	MIC/4	**32** (**0.5**)^S^	8 (1)^I^	>64 (≥1)^I^	>64 (≥1)^I^	>64 (≥1)^I^	<**0.5** (<**0.25**)^S^	<0.5 (≥1)^I^	4 (1)^I^	8 (1)^I^	<0.5 (≥1)^I^	20

TET	0	16	16	32	64	4	32	2	4	2	32	
	MIC/2	**8** (**0.5**)^S^	<**1** (<**0.062**)^S^	64 (2)^I^	**32** (**0.5**)^S^	<**0.5** (<**0.125**)^S^	**16** (**0.5**)^S^	**1** (**0.5**)^S^	<**0.5** (<**0.125**)^S^	2 (1)^I^	32 (1)^I^	**70**
	MIC/4	**8** (**0.5**)^S^	<**1** (<**0.062**)^S^	64 (2)^I^	**32** (**0.5**)^S^	<**0.5** (<**0.125**)^S^	32 (1)^I^	2 (1)^I^	4 (1)^I^	2 (1)^I^	32 (1)^I^	40

ERY	0	128	8	128	256	8	16	4	16	16	16	
	MIC/2	128 (1)^I^	**4** (**0.5**)^S^	**16** (**0.5**)^S^	**128** (**0.5**)^S^	8 (1)^I^	**4** (**0.5**)^S^	4 (1)^I^	16 (1)^I^	16 (1)^I^	**8** (**0.5**)^S^	**50**
	MIC/4	128 (1)^I^	8 (1)^I^	128 (1)^I^	**128** (**0.5**)^S^	8 (1)^I^	16 (1)^I^	4 (1)^I^	16 (1)^I^	16 (1)^I^	**8** (**0.5**)^S^	20

KAN	0	128	4	8	32	32	32	<2	16	16	8	
	MIC/2	128 (1)^I^	4 (1)^I^	**4** (**0.5**)^S^	32 (1)^I^	**16** (**0.5**)^S^	**8** (**0.25**)^S^	<2 (≥1)^I^	16 (1)^I^	**4** (**0.25**)^S^	8 (1)^I^	40
	MIC/4	128 (1)^I^	4 (1)^I^	8 (1)^I^	32 (1)^I^	**16** (**0.5**)^S^	**16** (**0.5**)^S^	<2 (≥1)^I^	16 (1)^I^	16 (1)^I^	8 (1)^I^	20

STP	0	256	>256	>256	128	8	64	8	16	>256	64	
	MIC/2	**64** (**0.25**)^S^	**256** (<**1**)^S^	>256 (≥1)^I^	128 (1)^I^	**4** (**0.5**)^S^	64 (1)^I^	**4** (**0.5**)^S^	16 (1)^I^	**256** (<**1**)^S^	**32** (**0.5**)^S^	**60**
	MIC/4	**64** (**0.25**)^S^	>256 (≥1)^I^	>256 (≥1)^I^	128 (1)^I^	8 (1)^I^	64 (1)^I^	8 (1)^I^	16 (1)^I^	>256 (≥1)^I^	**32** (**0.5**)^S^	20

AMP	0	>256	>256	>256	>256	>256	>256	>256	>256	>256	>256	
	MIC/2	**256** (<**1**)^S^	>256 (≥1)^I^	>256 (≥1)^I^	>256 (≥1)^I^	>256 (≥1)^I^	>256 (≥1)^I^	**128** (<**0.5**)^S^	>256 (≥1)^I^	**256** (<**1**)^S^	>256 (≥1)^I^	30
	MIC/4	>256 (≥1)^I^	>256 (≥1)^I^	>256 (≥1)^I^	>256 (≥1)^I^	>256 (≥1)^I^	>256 (≥1)^I^	>256 (≥1)^I^	>256 (≥1)^I^	>256 (≥1)^I^	>256 (≥1)^I^	00

CEF	0	>256	>256	>256	>256	>256	>256	>256	>256	>256	>256	
	MIC/2	>256 (≥1)^I^	>256 (≥1)^I^	>256 (≥1)^I^	>256 (≥1)^I^	**128** (<**0.5**)^S^	>256 (≥1)^I^	>256 (≥1)^I^	>256 (≥1)^I^	>256 (≥1)^I^	>256 (≥1)^I^	10
	MIC/4	>256 (≥1)^I^	>256 (≥1)^I^	>256 (≥1)^I^	>256 (≥1)^I^	>256 (≥1)^I^	>256 (≥1)^I^	>256 (≥1)^I^	>256 (≥1)^I^	>256 (≥1)^I^	>256 (≥1)^I^	00

^a^Antibotics [TET: tetracycline, CIP: ciprofloxacin, STP: streptomycin, KAN: kanamycin, CHL: chloramphenicol, ERY: erythromycin, AMP: ampicillin, and CEF: cefepime]. ^b^Bacterial strains: *Escherichia coli* [AG102, AG100Atet], *Pseudomonas aeruginosa* [PA124], *Enterobacter aerogenes* [CM64, EA27, EA289], *Klebsiella pneumonia* [KP55, KP63], *Providencia stuartii* [NAE16], and *Enterobacter cloacae* [BM47]. ^c^PBSS: percentage of bacteria strain on which synergism has been observed. Values in parenthesis are fractional inhibitory concentration (FIC) of the antibiotics after association with plants extract; S: synergy; I: indifference. The values in bold represent the cases of synergy between extract and antibiotic.

## Abbreviations

AMP: Ampicillin
ATCC: American type culture collection
CEF: Cefepime
CFU: Colony forming unit
CHL: Chloramphenicol
CIP: Ciprofloxacin
DMSO: Dimethylsulfoxide
EPI: Efflux pump inhibitor
ERY: Erythromycin
FIC: Fractional inhibitory concentration
INT: p-Iodonitrotetrazolium chloride
KAN: Kanamycin
MDR: Multidrug-resistant
MHB: Mueller-Hinton Broth
MIC: Minimal inhibitory concentration
PAßN: Phenylalanine-arginine-ß-naphthylamide
RND: Resistance nodulation-cell division
STR: Streptomycin
TET: Tetracycline.
