# Supplementary material for: Antibacterial and Antibiotic-Modifying Activity of Methanol Extracts from Six Cameroonian Food Plants against Multidrug-Resistant Enteric Bacteria
Source: Biomed Res Int. 2017 Aug 20;2017:1583510. doi: 10.1155/2017/1583510 (PMC5585599; doi:10.1155/2017/1583510)
Supplement: Supplementary file 1 — Table S 1. Preliminary test of MIC of different antibiotics after the association of the extracts at MIC/2, MIC/4, MIC/8 and MIC/16 against P. aeruginosa PA124 strain. [file 1583510.f1.pdf]

Table S 1. Preliminary test of MIC of different antibiotics after the association of the extracts at MIC/2, MIC/4, MIC/8 and MIC/16 against *P. aeruginosa* PA124 strain.

ATB	CON	*Citrus sinensis*				*Psidium guajava (leaves)*				*Persea Americana (bark)*				*Persea americana (leaves)*			
		**MIC/2**	**MIC/4**	**MIC/8**	**MIC/16**	**MIC/2**	**MIC/4**	**MIC/8**	**MIC/16**	**MIC/2**	**MIC/4**	**MIC/8**	**MIC/16**	**MIC/2**	**MIC/4**	**MIC/8**	**MIC/16**
**CHL**	**128**	**32(0,25)^{S}**	128(1)^{I}	128(1)^{I}	128(1)^{I}	128(1)^{I}	128(1)^{I}	128(1)^{I}	128(1)^{I}	**64(0,5)^{S}**	**64(0,5)^{S}**	**64(0,5)^{S}**	**64(0,5)^{S}**	**64(0,5)^{S}**	**64(0,5)^{S}**	**64(0,5)^{S}**	128(1)^{I}
**CIP**	**64**	**16(0,25)^{S}**	**32(0,5)^{S}**	**32(0,5)^{S}**	**32(0,5)^{S}**	**32(0,5)^{S}**	**32(0,5)^{S}**	**32(0,5)^{S}**	**32(0,5)^{S}**	**32(0,5)^{S}**	**32(0,5)^{S}**	**32(0,5)^{S}**	**32(0,5)^{S}**	64(1)^{I}	64(1)^{I}	64(1)^{I}	64(1)^{I}
**TET**	**16**	**4(0,25)^{S}**	**8(0,5)^{S}**	**64(4)^{A}**	**64(4)^{A}**	**8(0,5)^{S}**	**8(0,5)^{S}**	**8(0,5)^{S}**	**8(0,5)^{S}**	**8(0,5)^{S}**	**8(0,5)^{S}**	**8(0,5)^{S}**	**8(0,5)^{S}**	16(1)^{I}	16(1)^{I}	16(1)^{I}	**32(2)^{A}**
**ERY**	**128**	128(1)^{I}	128(1)^{I}	128(1)^{I}	128(1)^{I}	128(1)^{I}	128(1)^{I}	128(1)^{I}	128(1)^{I}	128(1)^{I}	128(1)^{I}	128(1)^{I}	128(1)^{I}	128(1)^{I}	128(1)^{I}	128(1)^{I}	128(1)^{I}
**KAN**	**128**	128(1)^{I}	128(1)^{I}	128(1)^{I}	128(1)^{I}	128(1)^{I}	128(1)^{I}	128(1)^{I}	128(1)^{I}	128(1)^{I}	128(1)^{I}	128(1)^{I}	128(1)^{I}	**64(0,5)^{S}**	**64(0,5)^{S}**	**64(0,5)^{S}**	128(1)^{I}
**STR**	**256**	**32(0,125)^{S}**	**128(0,5)^{S}**	**128(0,5)^{S}**	**128(0,5)^{S}**	**128(0,5)^{S}**	**128(0,5)^{S}**	**128(0,5)^{S}**	**128(0,5)^{S}**	**64(0,25)^{S}**	**64(0,25)^{S}**	**64(0,25)^{S}**	**64(0,25)^{S}**	**64(0,25)^{S}**	**64(0,25)^{S}**	**64(0,25)^{S}**	**64(0,25)^{S}**
**AMP**	**>256**	>256(≥1)^{I}	>256(≥1)^{I}	>256(≥1)^{I}	>256(≥1)^{I}	>256(≥1)^{I}	>256(≥1)^{I}	>256(≥1)^{I}	>256(≥1)^{I}	>256(≥1)^{I}	>256(≥1)^{I}	>256(≥1)^{I}	>256(≥1)^{I}	>256(≥1)^{I}	>256(≥1)^{I}	>256(≥1)^{I}	>256(≥1)^{I}
**CEF**	**>256**	>256(≥1)^{I}	>256(≥1)^{I}	>256(≥1)^{I}	>256(≥1)^{I}	>256(≥1)^{I}	>256(≥1)^{I}	>256(≥1)^{I}	>256(≥1)^{I}	>256(≥1)^{I}	>256(≥1)^{I}	>256(≥1)^{I}	>256(≥1)^{I}	>256(≥1)^{I}	>256(≥1)^{I}	>256(≥1)^{I}	>256(≥1)^{I}

ATB	CON	*Coula edulis*				*Mangifera indica (Leaves)*				*Camellia sinensis*			
		**MIC/2**	**MIC/4**	**MIC/8**	**MIC/16**	**MIC/2**	**MIC/4**	**MIC/8**	**MIC/16**	**MIC/2**	**MIC/4**	**MIC/8**	**MIC/16**
**CHL**	**128**	**64(0,5)^{S}**	**64(0,5)^{S}**	**64(0,5)^{S}**	**64(0,5)^{S}**	**4(0,0312)^{S}**	**8(0,062)^{S}**	**16(0,125)^{S}**	**32(0,25)^{S}**	**8(0,0625)^{S}**	**8(0,0625)^{S}**	**8(0,0625)^{S}**	**8(0,0625)^{S}**
**CIP**	**64**	**16(0,25)^{S}**	**16(0,25)^{S}**	**32(0,5)^{S}**	**32(0,5)^{S}**	**4(0,0625)^{S}**	**32(0,5)^{S}**	**32(0,5)^{S}**	**32(0,5)^{S}**	**32(0,5)^{S}**	**32(0,5)^{S}**	**32(0,5)^{S}**	64(1)^{I}
**TET**	**16**	16(1)^{I}	16(1)^{I}	16(1)^{I}	**32(2)^{A}**	**8(0,5)^{S}**	**8(0,5)^{S}**	**8(0,5)^{S}**	**8(0,5)^{S}**	**8(0,5)^{S}**	16(1)^{I}	16(1)^{I}	16(1)^{I}
**ERY**	**128**	128(1)^{I}	128(1)^{I}	128(1)^{I}	128(1)^{I}	**8(0,5)^{S}**	**64(0,5)^{S}**	**64(0,5)^{S}**	128(1)^{I}	128(1)^{I}	128(1)^{I}	128(1)^{I}	128(1)^{I}
**KAN**	**128**	128(1)^{I}	128(1)^{I}	128(1)^{I}	128(1)^{I}	**16(0,125)^{S}**	**32(0,25)^{S}**	**64(0,5)^{S}**	**64(0,5)^{S}**	**64(0,5)^{S}**	**64(0,5)^{S}**	**64(0,5)^{S}**	**64(0,5)^{S}**
**STR**	**256**	**64(0,25)^{S}**	**64(0,25)^{S}**	**64(0,25)^{S}**	**64(0,25)^{S}**	**128(0,5)^{S}**	**128(0,5)^{S}**	**128(0,5)^{S}**	**128(0,5)^{S}**	**64(0,25)^{S}**	**64(0,25)^{S}**	**64(0,25)^{S}**	**64(0,25)^{S}**
**AMP**	**>256**	>256(≥1)^{I}	>256(≥1)^{I}	>256(≥1)^{I}	>256(≥1)^{I}	**4(na)^{S}**	**256(na)^{S}**	>256(≥1)^{I}	>256(≥1)^{I}	>256(≥1)^{I}	>256(≥1)^{I}	>256(≥1)^{I}	>256(≥1)^{I}
**CEF**	**>256**	>256(≥1)^{I}	>256(≥1)^{I}	>256(≥1)^{I}	>256(≥1)^{I}	**4(na)^{S}**	**64(na)^{S}**	>256(≥1)^{I}	>256(≥1)^{I}	>256(≥1)^{I}	>256(≥1)^{I}	>256(≥1)^{I}	>256(≥1)^{I}

[a]Antibotics [TET: tetracycline, CIP: ciprofloxacin, STP: streptomycin, KAN: kanamycin, CHL: chloramphenicol, ERY: erythromycin, AMP: ampicillin, CEF: cefepime].

[b](): FIC (Fractional Inhibitory Concentration) of the antibiotics after association with plants extract; S: Synergy, I: Indifference; The values in bold represent the cases of synergy between extract and antibiotics
